# Adaptive feedforward control of closed orbit distortion caused by fast helicity-switching undulators

**DOI:** 10.1107/S160057752101047X

**Published:** 2021-10-21

**Authors:** Mitsuhiro Masaki, Hirokazu Maesaka, Kouichi Soutome, Shiro Takano, Takahiro Watanabe, Kouji Kubota, Takahiro Fujita, Hideki Dewa, Toru Fukui, Takashi Sugimoto, Masao Takeuchi, Hitoshi Tanaka

**Affiliations:** a Light Source Division, Japan Synchrotron Radiation Research Institute (JASRI), 1-1-1 Kouto, Sayo-cho, Sayo-gun, Hyogo 679-5198, Japan; b RIKEN SPring-8 Center, 1-1-1 Kouto, Sayo-cho, Sayo-gun, Hyogo 679-5148, Japan; c SPring-8 Service Co. Ltd, 1-20-5 Kouto, Shingu-cho, Tatsuno, Hyogo 679-5165, Japan

**Keywords:** adaptive feedforward, orbit control, helicity-switching undulator

## Abstract

A new correction algorithm for closed orbit distortion based on an adaptive feedforward control (AFC) has been developed. At SPring-8, two helicity-switching twin-helical undulators (THUs) had been implemented with conventional feedforward corrections. However, the validity of these corrections turned out to be expiring due to unforeseen variation in the error magnetic fields with time. The developed AFC system has been applied to the THUs dynamically updating the feedforward table without stopping the helicity switching amid user experiments. The error sources in the two THUs are successfully resolved and corrected even while the two THUs are switching simultaneously with the same repetition period. The actual operation of the new AFC system enables us to keep the orbit variations suppressed with an accuracy at the sub-micrometre level in a transparent way for light source users.

## Abstract 2

A new orbit correction scheme based on the adaptive feedforward control (AFC) is presented. The developed AFC technique provides a new option in orbit control for light source stabilization at synchrotron radiation facilities.

## 1. Introduction

Storage-ring-based light sources are now essential platforms for photon sciences and related applications including industrial purposes. One of the important figures of merit for light source users is the brightness of light, which is also related to the degree of transverse coherence. Light sources with orders-of-magnitude higher brightness and transverse coherence than third-generation light sources have been developed (Hettel, 2014 ▸; Tavares *et al.*, 2014 ▸; Biasci *et al.*, 2014 ▸) and such next-generation light sources with higher brightness are now opening a new era of future sciences and other applications (Chenevier & Joly, 2020 ▸). However, high stability as well as high brightness is essential for successful developments. In the modern light sources, the pointing stability of light should be significantly smaller than the beam size. This can be achieved by the closed orbit correction and other feedback controls (Rehm, 2013 ▸).

In some synchrotron light sources, twin-helical undulators (THUs) for fast photon helicity switching have been utilized in X-ray magnetic circular dichroism experiments at beamlines. The component devices of the THU are set to opposite helicities, and the light output to the experimental station in the beamline is switched by separating the two radiation cones spatially with static [ESRF (Elleaume, 1994 ▸), SLS (Schmidt *et al.*, 2001 ▸), BESSY II (Bahrdt *et al.*, 2001 ▸; Holldack *et al.*, 2020 ▸)] or dynamical [KEK-PF (Matsuba *et al.*, 2008 ▸; Harada *et al.*, 2010 ▸), SPring-8 (Hara *et al.*, 2003 ▸), SRRC (Lin *et al.*, 1998 ▸)] local electron-orbit bumps. In the case of fast dynamical orbit bumps with kickers operating, orbit perturbations to the whole storage ring due to the error kicks in the orbit bumps need to be carefully suppressed as much as possible, especially in the next-generation light sources.

Two THUs with fast kicker systems have been installed in the SPring-8 storage ring to provide helicity switching of soft X-rays: ID23 and ID25 (Hara *et al.*, 1998 ▸, 2003 ▸). The photon helicity is switched at 1 Hz for ID23, and at 1 or 0.1 Hz for ID25. Orbit variations due to periodic excitation of the kicker magnets on demand from user experiments have been observed for years, even though ID23 and ID25 are equipped with air-core-coil fast kicker magnets for feedforward corrections to suppress them. Periodic orbit fluctuations synchronized with the kicker excitation can gradually grow up to 10 µm (RMS) or more because of the deterioration with time of the feedforward correction accuracy.

One possible solution could be to integrate a so-called fast global orbit feedback system, which has not been equipped in the SPring-8 storage ring. However, the correction performance would be dominated by the resolution of collectors and the measurement accuracy of the beam position monitors (BPMs), since the error source itself to be corrected is not appropriately counteracted in that kind of global orbit correction scheme. In that sense, the global correction has an uncertain factor on the light source stability; it could bring a potential risk of causing unwanted local orbit distortions at insertion device (ID) sections, where the BPMs do not observe the orbit. Based on a fundamental orbit correction strategy to directly counteract an error source to be corrected, we have taken the following approach considering (i) the error sources are identified at the two THUs, (ii) the feedforward correction is already equipped, and (iii) the degradation of the correction is slow. We have introduced, instead of a conventional fast orbit feedback, a new closed orbit distortion (COD) correction algorithm based on adaptive feedforward control (AFC), in which the feedforward tables are dynamically updated. In our AFC system, we employ newly developed fast BPMs based on MTCA.4 (Maesaka *et al.*, 2019 ▸) to extract the orbit fluctuations from each ID with a high resolution and derive counter-kick patterns for updating the feedforward tables. The COD variations originating in the two error sources (ID23 and ID25) are independently suppressed by the new AFC system. Our goal for the orbit stabilization is to suppress the COD fluctuations of less than 1 µm RMS during the kicker excitation in a transparent manner where experimental users cannot observe any periodic disturbance.

In this paper, the existing THUs of SPring-8 for the optical helicity switching are briefly illustrated in Section 2. The AFC system developed to suppress the fast periodic orbit variations is described in Section 3. The derivation method of the counter-kicks for updating the feedforward correction table is given in Section 4. Experimental verifications of performance of the developed AFC system and results of long-term continuous operation are reported in Sections 5 and 6, respectively. The source point stability at each ID in SPring-8 achieved by the new AFC system and the influence on the correction accuracy of betatron tune variations during the user time operations are discussed in Section 7.

## 2. THUs with kicker system for periodic optical helicity switching in SPring-8

**Figure 1:**
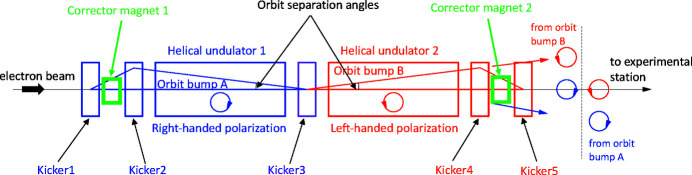
Schematic top view of the twin-helical undulator of SPring-8 with the horizontal kicker system for helicity switching. The orbit separation angles are 300 µrad and 100 µrad for ID23 and ID25, respectively.

**Figure 2:**
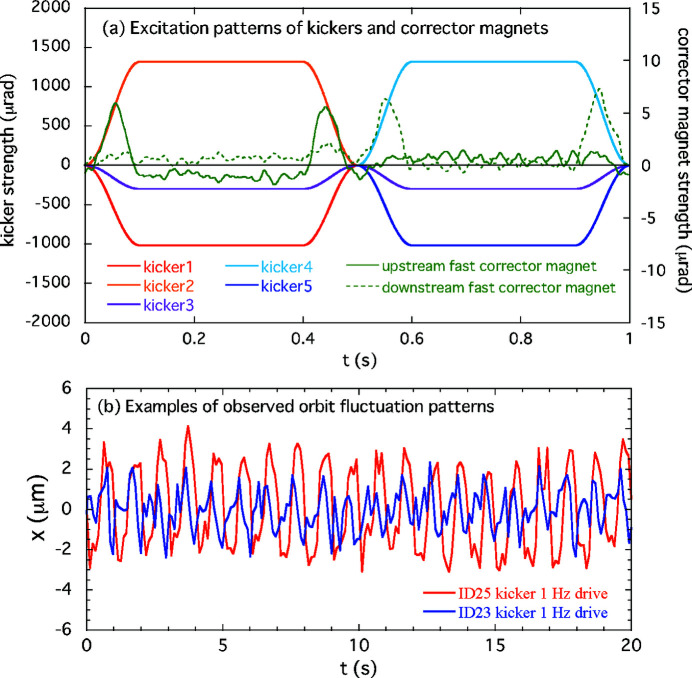
(*a*) Kicker excitation patterns and examples of excitation patterns for corrector magnets of ID23 for switching at 1 Hz. (*b*) Examples of periodic horizontal orbit fluctuations observed during solo 1 Hz helicity switching at ID23 and ID25.

Schematics of the THUs, ID23 and ID25, installed in the SPring-8 storage ring are illustrated in Fig. 1 ▸. The twin helical switching system consists of the two helical undulators for right- and left-handed circular polarizations, placed on the upstream and downstream sides, respectively. Five kicker magnets make the dynamical local horizontal orbit bumps (orbit A and B as shown in Fig. 1 ▸), for periodic optical helicity switching. The switching frequency for ID23 is 1 Hz; ID25 can be switched at either 1 or 0.1 Hz. When the electron beam orbit is switched to orbit A, the radiation from the upstream undulator travels horizontally off-axis and is stopped at the front-end absorber, whereas the radiation from the downstream undulator propagates along the beamline axis and reaches the experimental station. In the case of switching to orbit B, the situation is reversed. Thus, by switching these two orbits (A and B) repeatedly, the circularly polarized light with alternate helicities is provided for the user’s experiments. The photon beam separation angles for orbits A and B are 300 µrad for ID23 and 100 µrad for ID25. The five kicker magnets are excited with periodic trapezoidal patterns. As a typical example, the excitation pattern of ID23 kickers with a repetition frequency of 1 Hz is shown in Fig. 2 ▸(*a*). The kickers 1,2,3 making the orbit bump A are excited in the time range from 0 s to 0.5 s, whereas the kickers 3,4,5 making the orbit bump B are excited from 0.5 s to 1 s. To minimize the residual magnetic field integral for the three kickers producing the orbit bump, each set of the kickers 1,2,3 and 3,4,5 is connected in series with a separate power supply. The leakages of orbit bumps due to the kicker errors in the THU are supposed to be cancelled out by the feedforward control of the two fast air-core corrector magnets placed on both sides of the THU. The waveform-pattern signals to drive the power supplies for the kicker magnets and the fast corrector magnets are generated by a pattern board on a VME system which is prepared for each of ID23 and ID25 separately. The pattern board is equipped with 16-bits digital-to-analogue converters and outputs the waveform-pattern data sampled synchronously with an internal clock. The clock rate is set to 5 kHz or 1 kHz for the kicker switching frequency of 1 Hz or 0.1 Hz, respectively. While driving the kickers and the fast corrector magnets at 1 Hz or 0.1 Hz, the pattern board also provides outputs of a trigger signal synchronized with the pattern excitation.

The feedforward correction tables of fast corrector magnets for the optical helicity switching of ID23 and ID25 used to be updated a few times a year with the X-ray BPM (XBPM) data. The XBPMs (Aoyagi *et al.*, 2001 ▸) are photoelectron emission type with blade-type detectors that have been placed at the front-end of the ID beamlines. The existing data acquisition system of the XBPMs does not have enough time response for tuning the corrector magnets of the THUs, ID23 and ID25. The XBPMs were temporarily connected to a measurement device with a faster time response for that tuning. Therefore, the update of the correction tables was not able to be done timely during user time operation but rather during accelerator beam tuning time. However, as feedforward correction accuracy gradually degraded with time, we sometimes observed — for example, by using a BPM equipped with Libera Brilliance+ readout circuit (Instrumentation Technologies, 2021 ▸) — periodic orbit fluctuations in the horizontal plane, typically of several micrometres [see Fig. 2 ▸(*b*)] during the helicity switching of the THUs. The causes of this deterioration remain unclear, despite having been painstakingly investigated by the Insertion Device Group (Kinjo *et al.*, 2018 ▸). This led to the need for a new correction system that can automatically update the correction tables without stopping the necessary helicity switching during user experiments.

## 3. The AFC system suppressing the periodic orbit fluctuations for the helicity switching THUs

At SPring-8, in order to mitigate the problem of the periodic orbit perturbation while ID23 or ID25 is switching the optical helicities, we steered to the development of the AFC system to update the feedforward correction patterns dynamically and automatically. The AFC is equipped with dedicated fast BPMs with sufficient time response to detect the orbit variations precisely. In this section, the AFC system we developed to suppress the orbit fluctuation is described in detail.

Periodic COD fluctuation occurred in the horizontal plane synchronized to the kickers’ driving frequency of 1 or 0.1 Hz during the optical helicity switching at the two THUs. The observed periodic fluctuation contained frequency components up to several tens of Hz, but these are sufficiently slow compared with the radiation damping time of millisecond order. Therefore, we consider that the orbit distortion at each moment in the switching period can be analysed as a superposition of instantaneous CODs caused by time-dependent multiple error kicks at the kickers.

As shown in Fig. 1 ▸, each of the orbit bumps A and B is produced by exciting three kickers in a THU. Since the imperfect closure of the three-kicker orbit bump contains two error kicks, the leakage of COD variation outside the THU can be suppressed by adding two counter-kicks by exciting the two corrector magnets placed beside the kicker magnets in each THU. The betatron phase difference between the two THUs, ID23 and ID25, is approximately 660° in the horizontal direction, close to the ideal value (*i.e.* an odd multiple of 90°) for resolving the error sources relevant to each THU by observing the distribution of orbit distortion along the storage ring. Therefore, we decided to determine the values for the counter-kicks of the corrector magnets in the two THUs by measuring the orbit variation with multiple BPMs placed along the storage ring. The four counter-kicks for the two THUs can be approximately determined with the data of orbit distortion obtained by four BPMs placed on the ring by solving

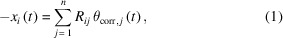

where *θ*_corr,*j*_(*t*) and *x*_*i*_(*t*) are the time-dependent counter-kick angle at the *j*th corrector magnet and the observed beam position displacement at the *i*th BPM, respectively. The response-matrix element between the kicker and the BPM is

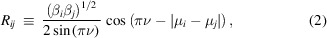

where the parameters β_*i*_, β_*j*_ are beta functions at the *i*th BPMs and the *j*th kickers; ν is the betatron tune; and μ_*i*_ − μ_*j*_ is the betatron phase advance between the kicker and the BPM, respectively.

**Figure 3:**
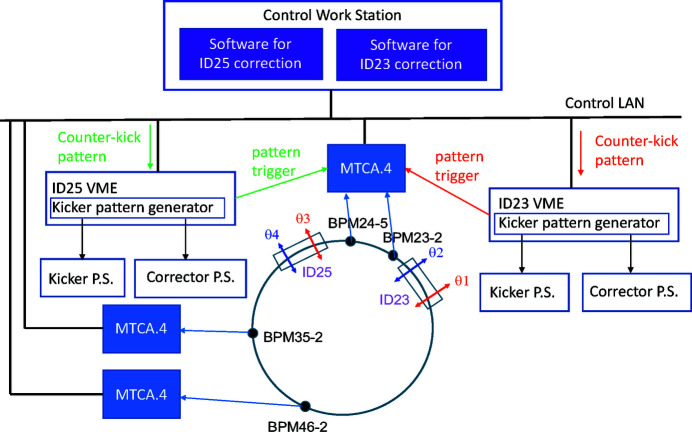
Overall picture of the adaptive feedforward control system at SPring-8.

Fig. 3 ▸ shows an overview of the AFC system at SPring-8. To resolve the four counter-kicks of the corrector magnets even while the two THUs are switching the helicity at the same frequency, we carefully selected four out of the total 288 BPM heads on the storage ring. Each of the selected BPM heads is connected to a newly developed fast MTCA.4-based readout circuit to detect the periodic COD variations with sufficient precisions. We selected two BPMs (namely, 23-2 and 46-2) sensitive to horizontal kicks in ID23, and two others (24-5 and 35-2) for ID25, considering the betatron phases between the IDs and the BPM heads. The calculated values of the response matrix elements between the selected BPMs and the fast corrector magnets for ID23/25 are listed in Table 1 ▸. The periodic COD variations during the kicker operations are measured by using the fast acquisition (FA) mode of the BPM circuit with a sampling rate of 10 kHz. The two trigger signals synchronized with the kicker driver for each THU, ID23 and ID25, are fed to a digitizer board on the common MTCA.4 system for BPM23-2 and 24-5. The other two BPM data sets are synchronized with the kickers by marking with the common time-stamps shared on the control network of the accelerator.

Our goal for orbit stabilization is to keep the COD fluctuations below 1 µm RMS even during the optical helicity switching. To achieve the orbit correction accuracy of less than 1 µm RMS, the kick errors of the fast corrector magnets need to be within 0.05 µrad (1σ). Sub-micrometre resolution is required for BPMs to detect periodic COD variations during the helicity switching. The fluctuation of the raw FA mode BPM data at the 10 kHz sampling is a few micrometres including random fluctuation of the electron beam itself in the horizontal direction. To obtain a sufficient resolution for resolving the counter-kicks at the corrector magnets precisely, BPM data are accumulated for several tens of periods of the kicker switching, folded at the period of the kicker, and numerically low-pass-filtered with a cut-off frequency of 100 Hz or less to reduce the high-frequency noise (see Section 5.1). After the process of folding and filtering the FA-BPM data, the 1σ resolution is expected to be approximately 0.2 µm including the intrinsic random noise in the BPM. The counter-kick data for updating the correction tables for the corrector magnets are calculated from equation (1) by using the processed BPM data with an improved signal-to-noise ratio. The new correction tables are obtained by adding the counter-kick data to the previous tables, which are written to memory banks on the VME pattern boards. An automatic update of the correction tables at appropriate time intervals will then ensure that the periodic orbit fluctuations continue to be suppressed.

## 4. Counter-kick calculation for the corrector magnets with singular value decomposition

In our AFC system, counter-kick patterns for the four corrector magnets in the two THUs, ID23 and ID25, are calculated using data from the four BPMs by solving equation (1) with the singular value decomposition (SVD) method. The 4-by-4 response matrix *R*_*ij*_ in equation (1) was obtained experimentally by measuring the four BPM responses to a single kick at each fast corrector magnet (Table 2 ▸). The betatron phase relationship between the two THUs and the four BPM heads selected for the AFC somewhat deviates from the ideal condition to resolve each error in the two THUs as shown in Section 3. The correction accuracy including these effects was quantitatively evaluated as follows.

In the case of the solo helicity switching of ID23 or ID25, counter-kick patterns for the two corrector magnets for each of ID23 and ID25 are calculated from the four-BPM data by solving equation (1) with SVD using two singular values (Table 3 ▸) of the 4-by-2 response matrix, in other words, multiplying by the pseudo-inverse response matrix. The errors in the solved counter kicks coming from the BPM error of 0.2 µm (1σ) are about 0.03 µrad (1σ) for both ID23 and ID25, which satisfies the target correction kick accuracy of 0.05 µrad (1σ) or less.

In the case of the simultaneous helicity switching of ID23 and ID25, the corresponding singular values of the 4-by-4 response matrix are shown in Table 4 ▸, in descending order from four modes #1 to #4. Solving equation (1) with the SVD using all four modes is equivalent to simply multiplying the inverse of the response matrix. Calculated CODs (RMS of value at the four BPMs) corresponding to each mode excited by a random kick of 0.1 µrad (1σ) at each corrector magnet are shown in Table 5 ▸. The CODs of modes #1 and #2 are the main components of the orbit variation and are larger than that of the modes #3 and #4. The CODs of modes #1 and #2 apparently need to be corrected. Modes #3 and #4 have significantly smaller contributions to the orbit variation. However, solving by using only modes #1 and #2 resulted in calculated counter-kicks with an inappropriate kick angle and sign. Comparing the BPM noise-derived errors of the counter kicks between the case where modes #1 through #3 except mode #4 are corrected (3-mode case) and the case where all four modes are corrected (4-mode case), the former is 0.05 µrad (1σ) and the latter is 0.1 µrad (1σ), indicating that the 4-mode case does not satisfy the target correction kick accuracy. Therefore, we decided to calculate the counter kicks in the 3-mode case (discarding mode #4).

## 5. Verifications

### 5.1. Folding and filtering processes of BPM data

The BPMs integrated into the new AFC system sample the orbit variations at 10 kHz. The waveform data acquired contain periodic orbit variations with the repetition rate (1 Hz or 0.1 Hz) of the switching kicker system. To reduce the influence of the BPM noise on the counter-kick calculation, the BPM data of several tens of periods in length are folded at the period of the helicity switching. In the case of helicity switching at 1 Hz, for example, the BPM data sampled at 10 kHz of 60 periods in length are firstly fast-Fourier-transformed (FFT) in the frequency range from −5 kHz to +5 kHz. We then pick up every peak accurately at the repetition frequency of the switching kicker and its harmonic frequencies by spline interpolation of the FFT spectrum. The inverse Fourier transform of the picked-up data at the fundamental and harmonic frequencies yields the time profile of periodic orbit variation folded at the period of the helicity switching. In the case of helicity switching at 0.1 Hz, the BPM data of 10 periods in length is folded by a similar procedure. For reducing the noise in the higher-frequency range, the fifth-orders Butterworth numerical low-pass filter is applied to data in the frequency domain before the inverse Fourier transform. The cut-off frequency is 100 Hz and 30 Hz for switching at 1 Hz and 0.1 Hz, respectively. By inversely Fourier transforming the lowpass-filtered peak data at the fundamental and harmonic frequencies of the switching, we can obtain an averaged and smoothed time profile of the periodic orbit variation at the switching frequency. The resolution of the processed BPM data due to the BPM random noise can be as small as 0.2 µm (1σ). Since the horizontal BPM data contain the random fluctuations of the electron beam orbit itself, the horizontal resolution of the processed BPM data was estimated by evaluating the fluctuations of the vertical BPM data (see Fig. 8) measured with the helicity switching halted. The fluctuation (resolution) of the vertical BPM data was about 0.22 µm (1σ) after processing by the same procedure for the horizontal data. Converting to the horizontal direction with the ratio of the position sensitivity coefficients of the BPM head in the horizontal and vertical directions, the resolution of the processed BPM is evaluated to be 0.15 µm (1σ) smaller than the requirement of 0.2 µm (1σ). In the processing of the BPM data, the frequency component at 0 Hz or the DC component was eliminated, since any residual COD is static and can be corrected by the global COD correction of the storage ring.

### 5.2. Verification of the correction scheme

**Figure 4:**
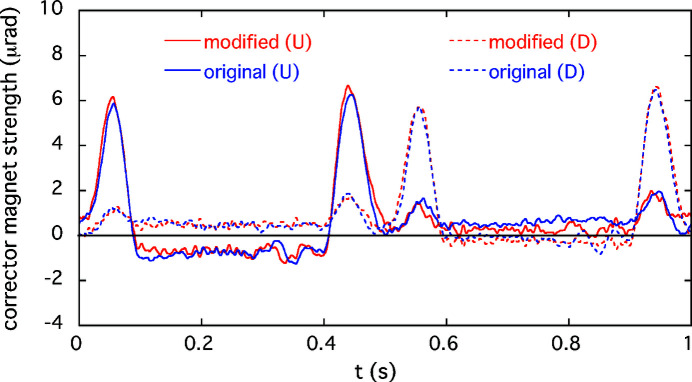
Modified (red) and original (blue) feedforward excitation patterns for the corrector magnets of ID23. The difference between the modified and original patterns is less than 1 µrad. The pole gap is 15.3 mm. (U: upstream; D: downstream.)

**Figure 5:**
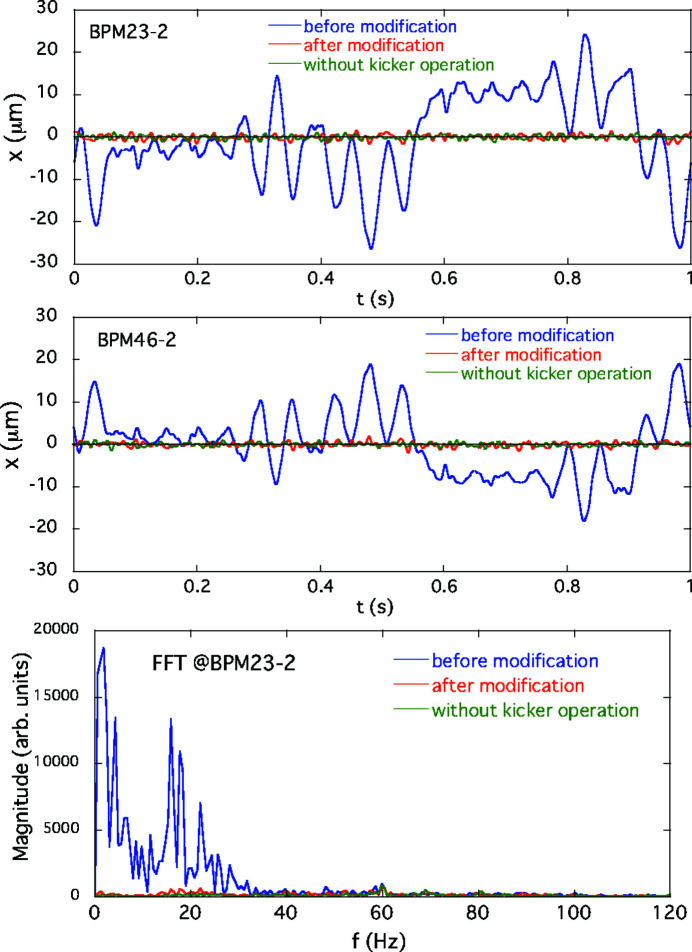
Horizontal periodic orbit fluctuations for the solo helicity switching of ID23 at 1 Hz observed by BPM23-2 and 46-2 compared before (blue) and after (red) modification of the excitation patterns for the corrector magnets. The green line shows the fluctuation without the helicity switching. Frequency spectra for the BPM23-2 data are also shown.

**Figure 6:**
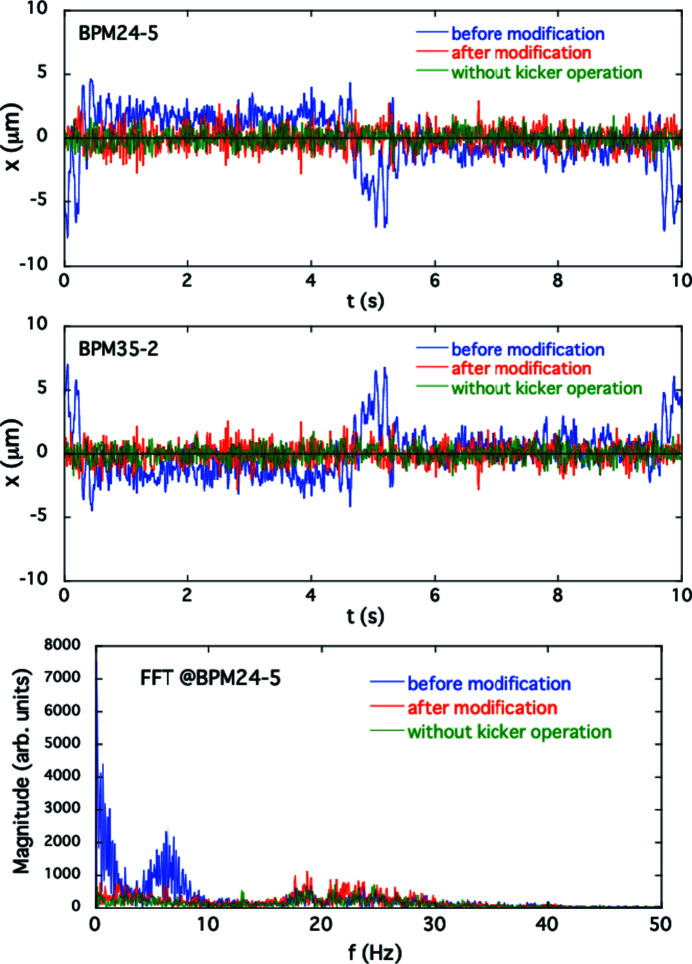
Horizontal periodic orbit fluctuations for the solo helicity switching of ID25 at 0.1 Hz observed by BPM24-5 and 35-2 compared before (blue) and after (red) modification of the excitation patterns for the corrector magnets. The green line shows the fluctuation without the helicity switching. Frequency spectra for the BPM24-5 data are also shown.

The performance of the AFC system for suppressing the periodic COD variation during the optical helicity switching was verified for both single and simultaneous switching at ID23 and ID25. As examples of the single-switching case described in Section 4, we take the corrections for the 1 Hz switching of ID23 and the 0.1 Hz switching of ID25. In the experiment for the first case of ID23, the initial periodic patterns in the feedforward correction tables for the corrector magnets shown in Fig. 2 ▸(*a*) still generated horizontal periodic COD fluctuation. Counter-kick data for updating the feedforward correction tables were obtained at several pole gaps of the ID23 by following the procedures in the AFC system mentioned above. An example of modified correction patterns for ID23 at a pole gap of 15.3 mm is shown in Fig. 4 ▸, along with the initial correction patterns. The periodic orbit fluctuation patterns obtained by processing the BPM data and the corresponding frequency spectra are shown in Fig. 5 ▸. After modifying the correction patterns, those orbit variations observed at BPM23-2 and 46-2 (the BPMs sensitive to the error kicks at ID23) are reduced to almost the same level as without helicity switching. In the frequency domain, we can see that the main components of the oscillation below 40 Hz are drastically damped. The correction tests of the AFC system for ID23 at other pole gaps also showed good results. Another example for the case of helicity switching of ID25 at 0.1 Hz is shown in Fig. 6 ▸. At BPM24-5 and 35-2 (sensitive to the error kicks at ID25), the orbit fluctuations of several micrometres were observed; after modification of the correction patterns, they were comparable with the level when the kicker was turned off. The frequency domain data also show that the main oscillation components below 10 Hz are well corrected.

**Figure 7:**
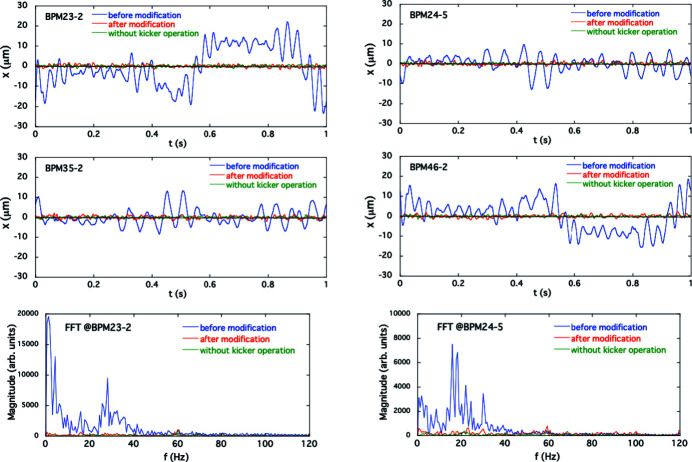
Horizontal periodic orbit fluctuations for the simultaneous helicity switching of ID23 and ID25 at 1 Hz observed by the BPMs compared before (blue) and after (red) modification of the excitation patterns for the corrector magnets. The green line shows the fluctuation without the helicity switching. Frequency spectra for the BPM23-2 and 24-5 data are also shown.

**Figure 8:**
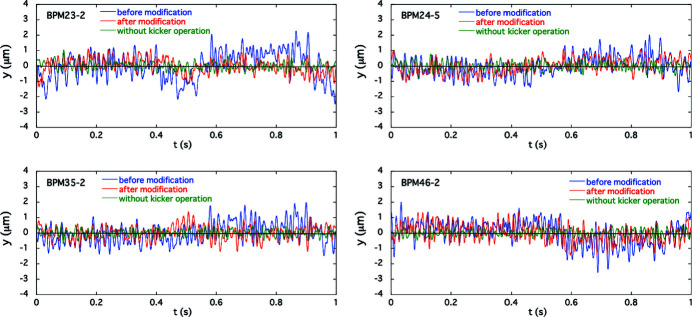
Vertical periodic orbit fluctuations for the simultaneous helicity switching of ID23 and ID25 at 1 Hz observed by the BPMs compared before (blue) and after (red) modification of the excitation patterns for the corrector magnets. The green line shows the fluctuation without the helicity switching.

In the experiment for simultaneous 1 Hz switching of ID23 and ID25, orbit fluctuations were detected at all four BPMs with the initial correction patterns in the feedforward correction tables for the two THUs. Following the procedure described in Section 4, we obtained the counter-kick data for updating the feedforward correction tables for both IDs. The calculation of counter-kick data for each ID was separately processed by using all four BPM data synchronized with the respective kicker triggers. The observed periodic orbit variations before and after modifying the correction tables, along with the frequency-domain orbit fluctuation, are shown in Fig. 7 ▸, in which the periodic COD variations were folded at the period of the kicker trigger for ID23. The pole gaps were 20 mm and 60 mm for ID23 and ID25, respectively. After the modification of the feedforward correction table, the horizontal COD fluctuations were reduced to the background level without helicity switching, as in the single-drive case. We also checked for adverse effects of the horizontal corrections on the vertical COD as those could be induced by, for example, tilted magnetic field in the fast corrector magnets due to fabrication and installation errors. The horizontal corrections worked well without compromise on the vertical beam stability as shown in Fig. 8 ▸. We thus verified that our new COD correction scheme based on the AFC was successful for both the single and simultaneous switching in the THUs.

## 6. Long-term performance of the AFC

**Figure 9:**
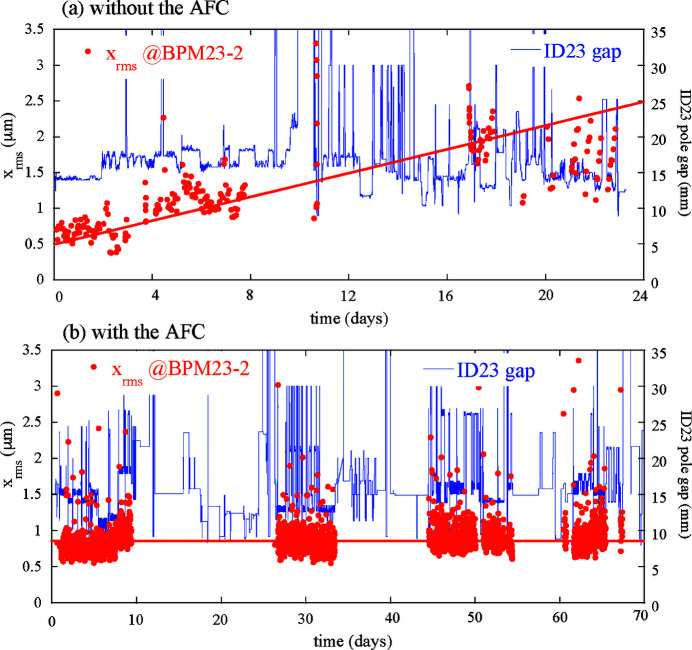
Example of RMS in the repetition period of helicity switching of the horizontal orbit fluctuations observed by BPM23-2 (red dots), (*a*) without and (*b*) with the continuous adaptive feedforward control (AFC). Without the AFC, the RMS fluctuation increased monotonically at about 80 nm day^−1^ for this time period (red line). Blue lines show the pole gap of ID23.

**Figure 10:**
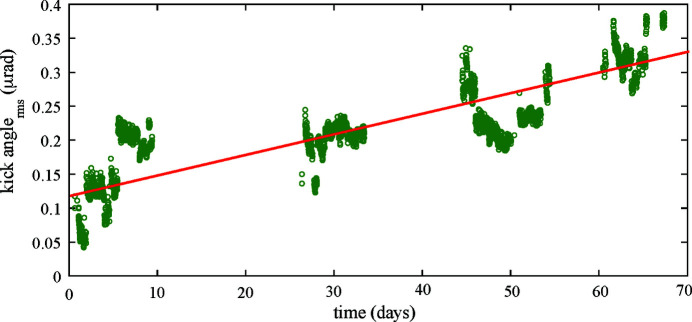
Example of history of RMS in the repetition period of helicity switching of the sum of kick angles for the up- and downstream corrector magnets (green dots) and a linear growth at a rate of about 3 nrad day^−1^ (red line) with the continuous adaptive feedforward control for the same time period as that of Fig. 12(*b*).

We applied the developed AFC to the helicity switching of ID23 during the user operation to confirm its long-term performance in suppressing the periodic COD variations. The helicity switching of ID25 was suspended at the time because of certain mechanical problems. The feedforward correction patterns were automatically updated at 10 min intervals with the AFC system. Long-term trends in the periodic orbit-fluctuation amplitude with and without the AFC are compared for the single switching of ID23 at 1 Hz. Fig. 9 ▸ shows the RMS-fluctuation trend of the periodic COD patterns (chosen as an indicator of the magnitude of the periodic orbit fluctuation at 1 Hz) observed by BPM23-2, together with the pole gap of ID23. The case without the AFC shows gradual growth of the fluctuation at a rate of approximately 80 nm day^−1^, indicating deterioration of the correction accuracy over time [see Fig. 9 ▸(*a*)]. With the AFC, by contrast, the orbit variation was successfully suppressed [see Fig. 9 ▸(*b*)]. The orbit fluctuation jumped up just after the ID23 pole gap changed, but it was promptly reduced by a subsequent correction by the AFC system. Fig. 10 ▸ shows the RMS trend of the sum of the periodic kick angles at the up- and downstream corrector magnets of ID23 during the continuous AFC operation. This indicates a growth rate of approximately 3 nrad day^−1^. Multiplied by the response function of ∼26 m rad^−1^ between ID23 and BPM23-2 (see Table 2 ▸), the growth rate of the orbit fluctuation without the AFC is estimated to be 78 nm day^−1^ at BPM23-2, which is consistent with the observed trend shown in Fig. 9 ▸(*a*).

## 7. Discussion

### 7.1. Evaluation of ID source point stability

**Figure 11:**
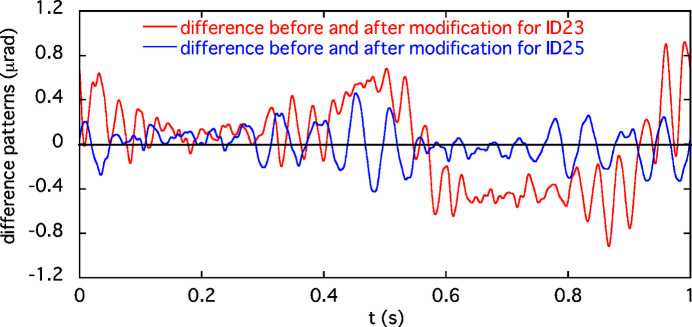
Differences before and after the modification in the sum of the correction patterns for the up- and downstream corrector magnets. Red and blue lines are for ID23 and ID25, respectively.

**Figure 12:**
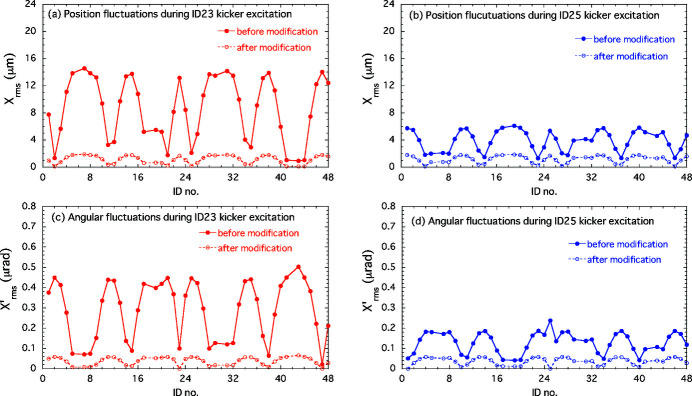
Comparisons of fluctuations of the ID sources in position and angle before and after the modification of excitation patterns of corrector magnets in cases of solo helicity switching at ID23 and ID25 at 1 Hz. Error kicks before the modification are assumed to be 0.38 µrad (RMS) for ID23 and 0.16 µrad (RMS) for ID25. The light source fluctuations after the modification are calculated values assuming the residual error kick of 0.05 µrad at ID23 or ID25, which is our target performance of the AFC correction.

We have estimated the impact of the AFC on the orbit stability at the ID light-source points over the storage ring. After the modifications of the feedforward correction patterns, the orbit fluctuations detected by the BPMs are suppressed near the noise level. Therefore, assuming that the error kicks at ID23 and ID25 are very small after the modifications, the residual kicks before the modifications can be approximately regarded as the differences between the correction patterns before and after the change. Using these residual kick patterns, the position and angular fluctuations (*x*_ID_, *x*_ID_′) at each ID source point are expressed by equations (3) and (4), respectively,

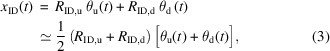




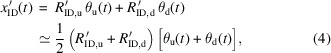

where θ_u_(*t*) and θ_d_(*t*) are the residual kick angles at the upstream and downstream corrector magnets, respectively. Similarly, (*R*_ID,u_, 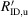 ) and (*R*_ID,d_, 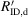 ) are the response matrices between the up/downstream corrector magnets and the IDs for the position and angular displacements, respectively. The betatron phase advance between the up- and downstream kickers is small owing to the large horizontal betatron function (∼30 m) at the ID23/25 sections. Therefore, the response matrices for both kickers are nearly equal and can be approximated by their average. An example of the differences in the sum θ_u_(*t*) + θ_d_(*t*), before and after modifying the correction-kick patterns, is shown in Fig. 11 ▸. The estimated position and angular fluctuations (RMS) at each ID source point before improving the corrections (using the RMS values 0.38 µrad and 0.16 µrad of the sum kick patterns for ID23 and ID25, respectively) are shown in Fig. 12 ▸. This is an example of a light source fluctuation corresponding to the orbit variation in the original correction patterns.

As described in Section 3, the kick error using the AFC correction is targeted to be below 0.05 µrad (1σ). The validation results in Section 5.2 show that the amplitudes of the orbit variations after the AFC correction were reduced to the background oscillation level for the kicker turned off. The residual kicks after the AFC correction, which were evaluated from the reduction rate of the orbit variations observed by the BPMs, were about one-tenth of 0.38 µrad and about one-third of 0.16 µrad for the ID23 and ID25 kicker drives, respectively, satisfying the target of 0.05 µrad. In Fig. 12 ▸, the light source fluctuations are shown for a typical error kick of 0.05 µrad after the AFC correction.

We now discuss the effect of the AFC on user experiments, in the case of the optical helicity switching of ID23, which has an orbit variation larger than that of the ID25 kicker. The angular fluctuation of light axis is at most 0.5 µrad (RMS) even in the ID with the largest angular variation, which is sufficiently small compared with a typical intrinsic photon divergence of an undulator of a few microradians, *e.g.* 3.3 µrad (RMS) for an X-ray wavelength of 1 Å and an ID length of 4.5 m. Therefore, the angular variation did not seem to hurt user experiments. On the other hand, the variation in the light source position is at most 14 µm (RMS), 5% or less of the electron beam size of 315 µm (RMS) at the source point. However, when a narrow slit in the beamline is used as a virtual light source and the X-ray is focused by a focusing device such as a Fresnel zone plate (FZP) *etc*., the angular variation of the light axis after passing through the slit can be several microradians on the focal plane where an experimental sample is placed. In the case of a distance *L* of 30 m from the ID source point to the slit and the source point fluctuation Δ*x*_s_ of 60 µm (peak-to-peak), the light angular fluctuation Δθ_s_ (= Δ*x*_s_/*L*) after passing through the slit is 2 µrad (peak-to-peak). This could be a hindrance, for example, in experiments such as measuring the X-ray scattering angles of a sample. By the AFC correction, the fluctuation of the light source position is reduced to approximately 1 µm (RMS) which is a level that does not affect the experiments.

### 7.2. Effect of betatron-tune variation on the correction accuracy

The horizontal tune of a storage ring can be varied due to ID gap changes during user operation. At SPring-8 the amount of horizontal tune variation is less than 0.01. The resulting deviations of the response matrix [equation (2)] between the fast corrector magnets and the BPMs most sensitive to each corrector are approximately 6–7% at most. The correction errors brought in by the errors in the counter kicks due to these deviations are sub-micrometre order, comparable with the correction errors due to the BPM noise. However, even if there are some kick errors due to the slight deviations in the response matrix caused by the betatron-tune variation, the variation is usually slow enough compared with the correction cycle of the AFC system. Therefore, any correction errors due to the deviations in the response matrix will be reduced by the successive corrections by the AFC and will not be a serious problem in practice.

## 8. Conclusion

A new COD correction technique with the AFC in a transparent manner for experimental users has been developed by using MTCA.4-based BPMs for suppressing fast periodic orbit fluctuations during operation of helicity-switching undulators with kickers. We verified at SPring-8 that the new method works well for orbit stabilization, even when the two THUs are switching optical helicity with the same repetition period. Operation of the AFC system keeps the COD fluctuation suppressed with sub-micrometre order for long times. The fluctuation is reduced to almost the same level as that without the helicity switching. The developed adaptive feedforward technique can be a powerful tool for electron orbit stabilization based on the error source suppression of deterministic disturbances with slow drifts in low-emittance synchrotron light sources. For the correction of this type of disturbance where error sources are identified, the developed AFC technique works very efficiently and effectively. In the coming next-generation light sources, an extreme pointing stability of the photon beam will be essential for taking full advantage of the new light source performances. The AFC will provide a new capability to implement the stability control, which we believe is essential to broaden the science opportunities on the future synchrotron light facilities.

## Figures and Tables

**Table 1:** Calculated response matrix *R*_*ij*_ between the BPMs selected for the AFC and the corrector magnets for ID23 and ID25

	ID23		ID25	
*R*_*ij*_ (m rad^−1^)	Upstream (θ_1_)	Downstream (θ_2_)	Upstream (θ_3_)	Downstream (θ_4_)
BPM23-2 (*x*_1_)	+26.0	+25.1	−3.90	+0.11
BPM24-5 (*x*_2_)	+0.083	−4.17	+25.1	+26.0
BPM35-2 (*x*_3_)	−1.66	+2.44	−23.0	−20.7
BPM46-2 (*x*_4_)	−18.7	−21.3	+5.44	+1.45

**Table 2:** Measured response matrix *R_*ij*_* between the BPMs selected for the AFC and the corrector magnets for ID23 and ID25

	ID23		ID25	
*R*_*ij*_ (m rad^−1^)	Upstream (θ_1_)	Downstream (θ_2_)	Upstream (θ_3_)	Downstream (θ_4_)
BPM23-2 (*x*_1_)	+26.4	+25.6	−3.07	+1.05
BPM24-5 (*x*_2_)	+0.623	−3.29	+25.6	+25.8
BPM35-2 (*x*_3_)	−2.08	+1.66	−22.8	−20.2
BPM46-2 (*x*_4_)	−18.2	−20.9	+6.69	+2.71

**Table 3:** Singular values of the measured 4-by-2 response matrix for calculating the two counter-kicks in the solo helicity switching of ID23 and ID25

Mode #	Singular values for ID23	Singular values for ID25
1	46.1	47.9
2	4.24	4.17

**Table 4:** Singular values of the measured 4-by-4 response matrix for calculating the four counter-kicks when ID23 and ID25 are simultaneously switching the helicity

Mode #	Singular values
1	50.4
2	43.7
3	1.89
4	1.22

**Table 5:** Calculated r.m.s. CODs corresponding to each mode at four BPMs when the kick angle of 0.1 µrad (1σ) is randomly given at each correction kicker

COD (µm)	Kick at ID23-U	Kick at ID23-D	Kick at ID25-U	Kick at ID25-D
Mode #1	0.94	1.2	3.9	1.3
Mode #2	1.3	1.2	0.84	1.0
Mode #3	0.05	0.04	0.05	0.06
Mode #4	0.03	0.04	0.03	0.02
